# Simulation of Mechanical Stresses in BaTiO_3_ Multilayer Ceramic Capacitors during Desoldering in the Rework of Electronic Assemblies Using a Framework of Computational Fluid Dynamics and Thermomechanical Models

**DOI:** 10.3390/ma17112702

**Published:** 2024-06-03

**Authors:** Adam Yuile, Erik Wiss, David Barth, Steffen Wiese

**Affiliations:** Chair of Microintegration and Reliability, Saarland University, 66123 Saarbrucken, Germany; adam.yuile@uni-saarland.de (A.Y.); erik.wiss@uni-saarland.de (E.W.); s8dabart@stud.uni-saarland.de (D.B.)

**Keywords:** MLCC, BaTiO_3_, reflow, desoldering, hot air rework, CFD, FEM, thermomechanical stresses

## Abstract

Multilayer ceramic capacitors (MLCCs) are critical components when thermal processes such as reflow desoldering are used during rework of electronic assemblies. The capacitor’s ferroelectric BaTiO_3_ body is very brittle. Therefore, thermomechanical stresses can cause crack formation and create conductive paths that may short the capacitor. In order to assess the thermally induced mechanical stresses onto an MLCC during reflow desoldering, simulations were carried out, which make use of a framework of computational fluid dynamics and thermomechanical models within the ANSYS software package. In the first step, CFD simulations were conducted to calculate the transient temperature field in the surrounding of the MLCC component, which was then used as an input for FEM simulations to compute the arising mechanical stresses inside the MLCC. The results of the simulations show that the major contribution to mechanical stresses within the MLCC component comes from the mismatch in thermal expansion between the printed circuit board and the MLCC. The temperature gradients along the MLCC component are rather small and account only for moderate internal stresses within the brittle BaTiO_3_ body.

## 1. Introduction

**Figure 1:**
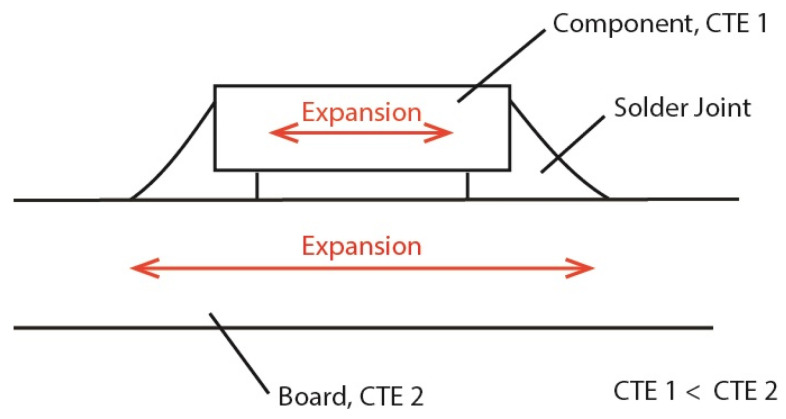
Sketch of an MLCC component soldered on a PCB. The mismatch in the thermal expansion coefficients leads to a different expansion of the MLCC and the PCB, which results in thermomechanical stress in the system. Due to this stress, cracks can occur in the solder joints or the component, resulting in failures.

Electronic devices are usually manufactured in a standardized architectural concept, which outlines the process sequences and the interconnect interfaces, in order to manage the complex assembly process which spans over orders of magnitude in the dimensions of individual structural elements and processes and includes a large number of different materials. After the manufacturing of single electronic components (e.g., ICs, capacitors, resistors) at the interconnect levels 0 and 1, the first functional electronic circuits are assembled in the second interconnect level by connecting the components on a substrate (e.g., PCB, flex, ceramic carrier) before these functional units are wired to the final devices or systems at the interconnect levels 3 to 5 [1,2,3,4,5,6]. Therefore, the second interconnect level marks the central assembly step, where the functionality of electronic circuits is brought to life. Similar to component manufacturing, it uses cohesive joining to provide mechanically robust interconnects with low inductance and high conductivity. The preferred joining method is soldering using tin-based solders with a low melting point between 138 °C and 280 °C. Soldering is usually employed to mount components on a rigid, organic printed circuit board (PCB). Niche applications use other substrates like flexible circuit boards and high-temperature-resistant ceramic substrates, where soldering is still dominant. Only temperature-sensitive substrates (e.g., thermoplastics), which make only a small part of electronics, use other joining materials, like electrically conductive adhesives. One of the big advantages of soldering electronic components on a PCB is the possibility of rework, when either a malfunctioning component is assembled or the electronics fail in service, because of unnoticed flaws in the employed components. One of the critical components in this respect are multilayer ceramic capacitors (MLCCs), which are based on the use of ferroelectric materials, such as BaTiO_3_. The brittleness of their ceramic bodies, combined with geometrical discontinuities at the metal terminals, makes MLCCs prone to cracking when they undergo larger bending stresses. Figure 1 schematically shows the mechanism that creates bending stresses in the MLCC/board assembly. Since the coefficient of thermal expansion (CTE) of the component is often significantly smaller than that of the PCB, the expansion of the component is smaller when the temperature increases. This difference results in a thermomechanical stress in the system, which can evoke cracks and, thus, lead to failures. Typical failures are, for instance, hot spots on the electronic assembly, when a power line with high current supply is connected to the capacitor [7]. Another reliability issue is the electrolytic migration of electrode material (e.g., Ag). The migration is caused by constant voltages across the capacitor in combination with higher levels of relative humidity [8,9,10,11]. The migrating electrode metal can build conductive paths along the cracks. This could cause catastrophic damage in the relevant area as a result of severe shorts through the capacitor.

To achieve a minimal thermal loading of sensitive electronic components but to also to make sure that solder joints are formed in the required quality, a number of experimental methods have been established. In order to better understand why soldering of electronic components sometimes fails, theoretical models and simulation approaches were developed. Early studies modeled heat conduction in solid structures using simple 2D- and 3D-finite element analysis [12,13]. Somewhat more refined models were used by later investigations in order to describe the flow of hot gas inside reflow ovens [14,15]. Other studies have focused on the heat transfer from the hot gaseous media to solid structures, like components or boards, as well as on the description of solidification of the solder material [16,17]. In this respect, a number of applied experimental methodologies were developed to cover the effect of latent heat, for the accurate description of melting and solidification of solder joints [18,19,20,21].

While most of the experimental und simulation studies have focused on the optimization of the standard reflow process for the assembly at the second interconnect level during the manufacturing of electronic devices, the usage of reflow soldering in other situations, such as the rework of populated printed circuit board, has not been under a wider consideration. However, with the increasing attention to reduce electronic waste, rework processes will become more and more important. Thus, a better understanding of potential thermomechanical damage of components rework needs to be developed.

## 2. Materials and Methods

### 2.1. Experimental

#### 2.1.1. Board and Components

MLCC components from two different manufacturers (KEMET C series and KYOCERA AVX) in size 0805 and 1206 were used for the experiments. The MLCC types KEMET C0805C684K5RACTU and C1206C474K5RACTU consisted of an X7R dielectric, whereas the MLCC types KYOCERA AVX 08055G104ZAT2A and 12065G105ZAT2A consisted of a Y5V dielectric. Except for the height, the dimensions of the components of each size are quasi-identical (0805: length 2 mm, width 1.25 mm; 1206: length 3.2 mm, width 1.6 mm). Concerning the height, the KEMET is thicker for the 0805 (1.25 mm vs. 1.02 mm) and thinner for the 1206 (0.9 mm vs. 1.27 mm). Table 1 summarizes the major technical data of the components.

**Figure 2:**
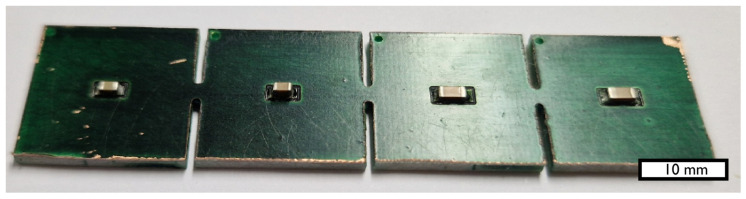
Test PCB with sliced areas for easier dicing after the experiments were conducted. The PCB was assembled using the following components: MLCCs of size 1206 and 0805 with various types of the dielectric (X7R, Y5V).

In order to be able to rebuild the modeled reflow desoldering process experimentally, a test PCB was designed and manufactured (see Figure 2). The thickness of the PCB’s FR-4 base material was 1.6 mm and the topside copper layer was 35 µm. Manufacturing of the PCB was realized on an LPKF Protomat S103 milling machine. The layout was kept simple and provides only soldering pads for different MLCC sizes. Each pair of these pads is separated in its own area so that they potentially can be diced easily, e.g., for microscopic analysis after reflow desoldering and resoldering.

After removing the oxide layers of the PCB surface with a rough pad, a sufficient amount of solder paste LOCTITE GC 10 SAC305T4 (Henkel) was deposited on the soldering pads using a compressed air dispenser DX-250 (OKI), and the MLCCs were placed using a pick and place system ProtoPlace S (LPKF). Finally, the soldering process was realized in a batch reflow oven ProtoFlow S (LPKF). According to the solder paste manufacturer, an appropriate reflow profile was chosen for this process.

#### 2.1.2. Rework Stations

Traditional rework was carried out manually using a standard soldering iron and a vacuum pump to remove solder from the pins of classical components made for through hole technology (THT). However, with the introduction of surface mount technology (SMT), the procedures in rework had to change, since the interconnect pads of surface mount devices (SMDs) were directly placed on the components body and no legs or wires were used anymore. Therefore, the use of a soldering iron induced a lot of heat to the functional part of the components. Thus, components as well as the PCB’s soldering pads were often significantly damaged by the transferred heat. Moreover, new package types were developed, which made the use of soldering irons impractical, because they consisted of heat-sensitive materials (e.g., LEDs) or contained hidden solder interconnections (e.g., BGAs). New equipment was developed, which was established in the form of a hot gas reflow soldering apparatus.

**Figure 3:**
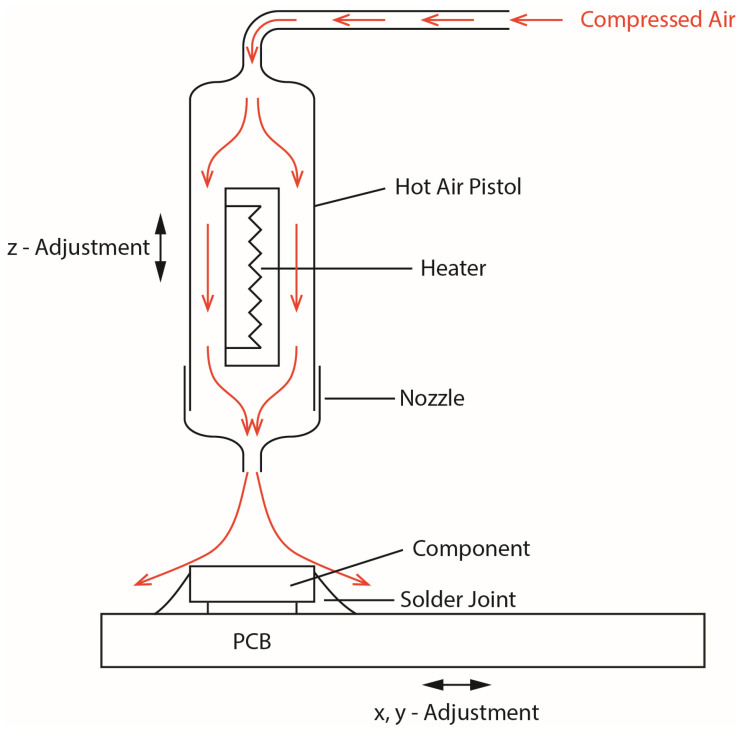
The hot air rework station uses compressed air that is heated up to a specific temperature. The air is blown into a hot air pistol which contains a heating element to heat the air. An exchangeable nozzle, whose size and form depends on the component that should be soldered or desoldered, is mounted at the outlet of the pistol.

**Figure 4:**
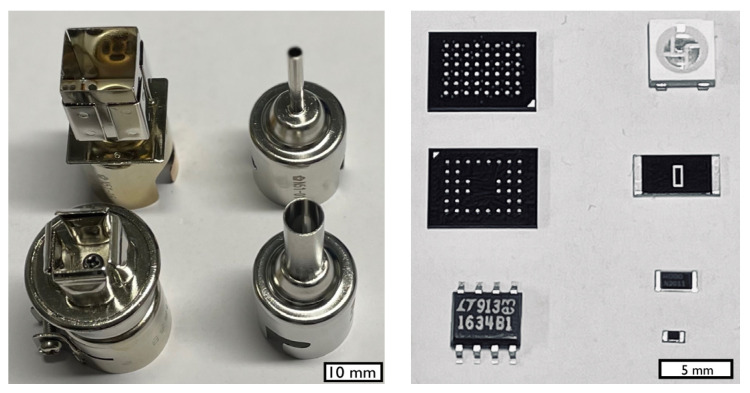
Different nozzles to adapt the hot gas jet to the size of the component (**left**). Typical sizes of SMT components, such as ICs (FBGA, SO), LED, 2512-, 1206-, 0603-SMT-resistors (**right**).

Figure 3 shows a schematic of the functional construction of a reflow soldering rework station. The central part is the hot air pistol which contains a temperature-controlled heating element and is supplied with compressed air. Both the temperature and the gas flow can be adjusted to receive optimum soldering results. Figure 4 shows different nozzles which help to adapt the airflow to the specific size of the component. The nozzle can be mounted on the outlet of the pistol.

**Figure 5:**
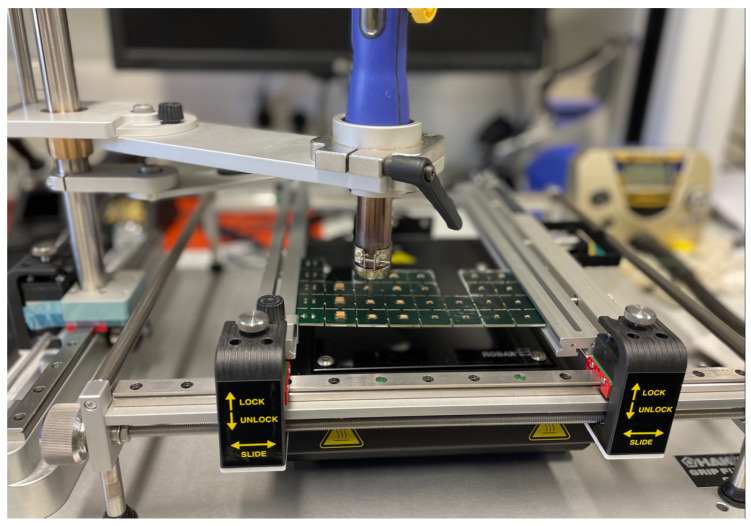
Overview photograph of the FR-811 (HAKKO Corp., Tokyo, Japan) rework station with mounted N51-01 nozzle. The hot air pistol is mounted on an adjustable grip over the board holder.

The rework station usually consists of a bottom heater and a board holder, to which the hot air pistol can be positioned to in x,y,z-direction. This way, the hot air jet can be directed to the component that should be desoldered from or resoldered to the PCB. Figure 5 shows an overview photograph of the used rework station.

Two different manual rework stations were used for the experiments. Both stations work on the principle depicted in Figure 3. The first station was an FR-811 (HAKKO Corp.), the second station was an 852-A (C.I.F.). The reason to use two different stations is caused by the different capabilities of the stations. The FR-811 is a more powerful station with a potential to provide a strong airflow. However, its sophisticated monitoring and control functions make experimenting sometimes difficult. In contrast, the 852-A is a very simple station with temperature and air flow adjustment. Both stations can carry the same nozzles. For all experiments, an N51-01 nozzle (HAKKO Corp.) with a round outlet having a diameter of 2.5 mm was used.

#### 2.1.3. Temperature Measurement

**Figure 6:**
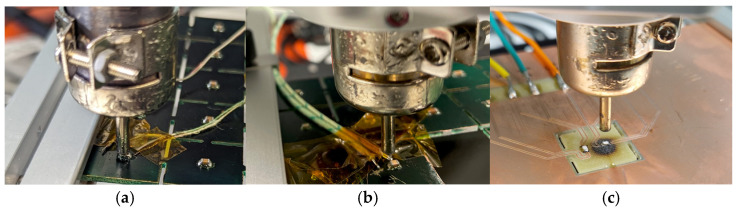
Configurations for temperature monitoring during desoldering experiments—(**a**) thermocouples, FR81; (**b**) thermocouples, 852-A, (**c**) PT-100, 852-A.

In order to monitor the temperature of the component during desoldering, different methods have been employed. The standard method is the use of thermocouples, which are mounted to the relevant objects using Kapton adhesive tape (Figure 6a,b). While the FR-811 rework station (Figure 6a) offers two thermocouple inputs for temperature control, monitoring and recording, the temperature recordings for the 852-A rework station (Figure 6b) were made using a DAQ6510 (Keithley) universal multimeter. As an alternative temperature monitoring method, SMT-component integrated PT100 sensors were employed (see Figure 6c).

### 2.2. Modeling

Computational fluid dynamics (CFD) models have become valuable tools for studying temperature distributions during soldering processes in electronic packaging. These models are particularly valuable for assessing the thermomechanical behavior of components such as multilayer ceramic capacitors (MLCCs) as they undergo the desoldering process from a printed circuit board (PCB).

**Figure 7:**
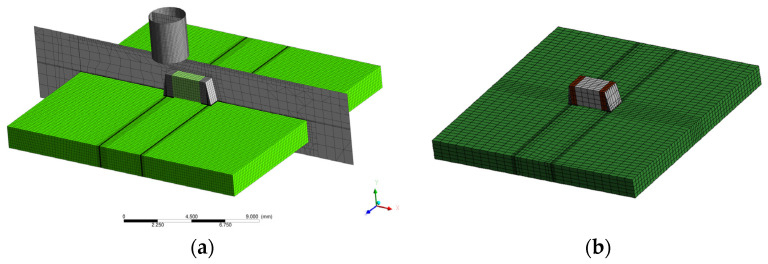
Geometrical models used for the simulations: (**a**) CFD model of 2.5 mm diameter nozzle displaced 1 mm directly above the left terminal of a 1206 MLCC. The model is depicted with the surface mesh and the mesh on the central plane, which is adapted for y^+^ during the solution. The surface mesh shows the fully structured base mesh with 5 adaptation cycles undertaken, with respect to the chosen y^+^ criterion, for refinement in the highly viscous near-wall regions. The display of the central plane provides an overview of the structure of the mesh in the air/atmospheric region, as well as between the nozzle exhaust and the component. (**b**) Corresponding mechanical model also having a fully structured hexagonal mesh for the mechanical submodel, with air and nozzle structures suppressed from initial CFD model. Temperature fields were imported from CFD mesh as inputs for mechanical modeling.

Given the context of simulating MLCC component removal, CFD models were constructed to simulate the complex thermodynamics within the disassembly procedure. The models consist of a PCB with copper pads and solder joints that accommodate an MLCC with connectors positioned on both sides, as shown in Figure 7. To mimic real-world conditions, a cylindrical nozzle is incorporated that produces a high-velocity jet at elevated temperatures, like that of a heat gun commonly used in manual resoldering or component removal for recycling purposes. Figure 7 shows the overall CFD model featuring a 1.25 mm radius nozzle displaced to the left side of the MLCC component, directly above the left terminal. The material data that were used to describe the thermal properties for the CFD simulations, which were mainly sourced from the ANSYS GRANTA material database, are summarized in Table 2.

In the CFD model, the following conservation equations were solved: continuity; energy; x-, y-, and z-momentum; and a two-equation SST k-Ω turbulence model. SIMPLE pressure–velocity coupling and second-order discretization schemes for all parameters in space and a first-order implicit transient formulation were also used. While the implementation of melting/solidification models, as it was, for instance, used in [22], would model the solder behavior more realistically, it comes with the cost of significantly increased computational times. Experience gained from earlier studies, e.g., reflow and wave soldering process simulation [23,24], showed that the implementation of phase change models was found to be very restrictive in terms of the permissible time-step size. Therefore, for the specific goals of this study, a less computationally intensive way was prioritized because the modeling of solidification would only be highly beneficial if the effect of an external force (e.g., from a gripper) needed to be analyzed.

The CFD model comprises essential components, including a nozzle, 1206 MLCC, and 35 µm thick copper pads designed for mounting on a 1.6 mm thick PCB of 18 × 18 mm^2^ in size. As the materials differ throughout the model, so too do the solid cell zones in ANSYS Fluent. After importing the mesh into Fluent, these adjacent cell zone interfaces are modified by slitting the interior between the different solids, leaving interior regions that allow for transport of energy through the interface between the dissimilar materials.

The distance between the exit of the nozzle and the top surface of the MLCC is set at 1 mm, reflecting typical operational conditions. To simulate real-world conditions accurately, an air atmosphere envelops these solid structures within the model. This approach aligns with established CFD methodologies.

These CFD models simulate the jet’s impact on the MLCC terminals by deliberately introducing an offset from the center. This deliberate asymmetry creates a temperature gradient across the MLCC that reflects practical scenarios. Such asymmetry could be critical as it allows assessment of potential failure mechanisms, including the risk of component cracking that may result from uneven thermal stresses created during the desoldering process.

**Figure 8:**
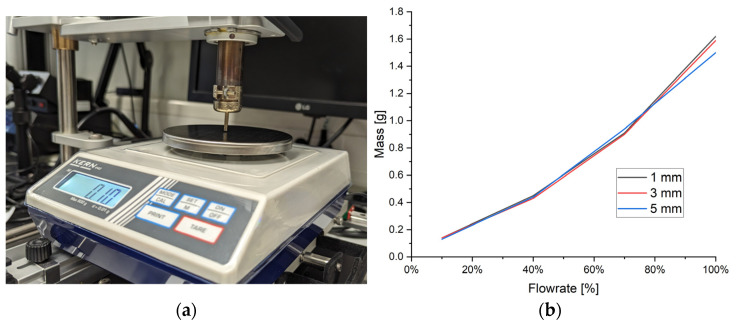
Calibration of flow velocity: (**a**) KERN weighing scales placed under nozzle with a displacement varying between 1 and 5 mm between the nozzle exit and the weighing pan; (**b**) measurements from KERN weighing scales for 1, 3, and 5 mm displacement between nozzle exit and weighing pan for 10, 40, 70, and 100% flowrate on FR-811 (HAKKO) device.

To establish accurate assessment of the flow velocities exiting the nozzle, measurements were performed by placing a weighing scale underneath the nozzle, as shown in Figure 8, and measuring the force generated by the exhausted jet. The flowrate percentages were varied between 10 and 100% on the HAKKO reworking tool, and, likewise, the displacement between the nozzle exit and the weighing pan was set to 1, 3, and 5 mm, where the results are shown in Figure 8a.

Firstly, it was observed for the lower flowrates that the displacement between the nozzle and weighing plan was not particularly significant, albeit this does start to become more significant at higher flowrates. With a displacement of 1 mm, the FR-811 (HAKKO) produced a measurement of 1.62 g on the weighing scales at 100% flowrate, as per Figure 8b. Having measured these forces, the CFD models were adapted (without the MLCC component) such that a pure jet impinged on a flat plate, for otherwise the same nozzle geometry, thus replicating conditions of Figure 7. Through this approach, it was found that an inlet velocity of 49.66 m/s produced the same net force measured on the weighing scales and, hence, this value was used for subsequent calculations presented here, because it is standard flow speed which is used for the majority of desoldering processes. Other flow speed variations were not studied, because they are generally linked to other types of components, which are either temperature-sensitive (e.g., LED plastic packages) or have large dimensions (e.g., BGA packages). Henceforth, velocity and temperature boundary conditions were set at 49.66 m/s and 400 °C, respectively, at the nozzle inlet. Atmospheric conditions were present at the outlet boundaries of the CFD domain, with reversed flow re-entering at a temperature of 300 K (26.85 °C).

The initial CFD mesh for the 1206 MLCC comprised 21,000 elements, which was then refined to 174,000 elements after the five refinement cycles based on a 50 < y^+^ < 500 mesh refinement criteria, ensuring reasonable resolution of the boundary layers. This step was crucial for accurately modeling heat transfer between the air and the solid structures. Subsequently, the entire temperature field, of fluid and solid structures, was reinitialized to room temperature (300 K), retaining the solved steady-state velocity field as initial conditions for the unsteady model. The temperature fields obtained from CFD were then utilized as inputs for a thermomechanical assessment of stresses induced by the hot air pistol in ANSYS Mechanical for a standard static structural analysis. The reference temperature, which represents the stress-free state, was taken to be room temperature. This assumption was made for practical reasons, because the true stress-free state depends on a larger number of unknown parameters, such as the manufacturing process of the MLCC, the state of aging of the assembly, the loading history of the solder joints, etc. To accurately account for all these factors would generate a not-proportional complexity of the used preconditions compared to other model assumptions implied for the mechanical simulation; thus, small modeling errors by the simple and not totally groundless assumption of a stress-free condition at room temperature were found to be acceptable.

The material properties for the thermomechanical FEM-simulations listed in Table 3 were sourced from the ANSYS GRANTA material database, and a bilinear kinematic hardening plasticity model was used for the solder. The solder model, which is characterized by 38 MPa and tangent modulus of 353 MPa, was taken from [25], from which the data published in [26] were adopted.

## 3. Results

### 3.1. Temperature Monitoring

**Figure 9:**
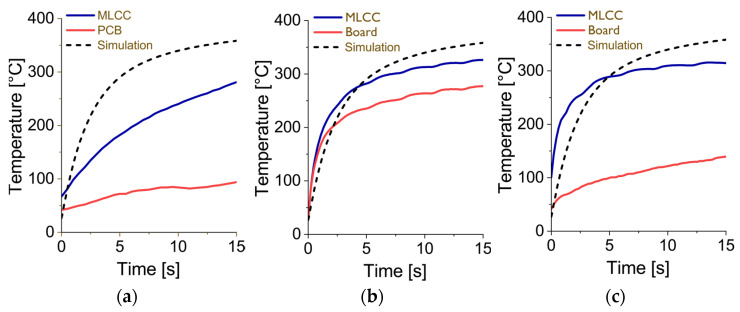
Simulated (dashed black line) and experimentally monitored temperatures of the MLCC body (blue line) and the PCB (red line) in the vicinity of the MLCC during the desoldering process using (**a**) the FR-811 rework station with thermocouples, (**b**) the CIF 852-A rework station with thermocouples, and (**c**) the CIF 852-A rework station with PT-100 sensors. The adjusted target temperature was 400 °C. The gas flow was set to medium intensity.

Figure 9a shows the monitored temperatures of the MLCC body (blue line) and the PCB (red line) in the vicinity of the MLCC during the desoldering process, when two K-type thermocouples were fixed on the MLCC and the board by Kapton adhesive tape. The thermocouples were connected to the FR-811 control unit, which recorded the temperatures during the desoldering process using thermocouples. The experimentally monitored curves are overlain by the results of the simulation for the MLCC body temperature (dashed black line).

The diagram in Figure 9b shows the monitored temperatures of the MLCC body (black) and the PCB (red) in the vicinity of the MLCC during the desoldering process, which was carried out by an 852-A rework station. The temperatures of two K-type thermocouples were recorded using a DAQ6510 (Keithley) universal multimeter. The same configuration was used to monitor the temperatures shown in the diagram in Figure 9c. Instead of the thermocouples two SMT-resistor integrated PT-100 sensors were employed. These sensors were directly deposited on the ceramic body of the SMT resistors, which were placed in such a way that the PT-100 structures were situated on the bottom side, with no direct contact to the hot gas jet. All diagrams in Figure 9 contain an overlay of the simulated values for comparison.

### 3.2. Simulation

**Figure 10:**
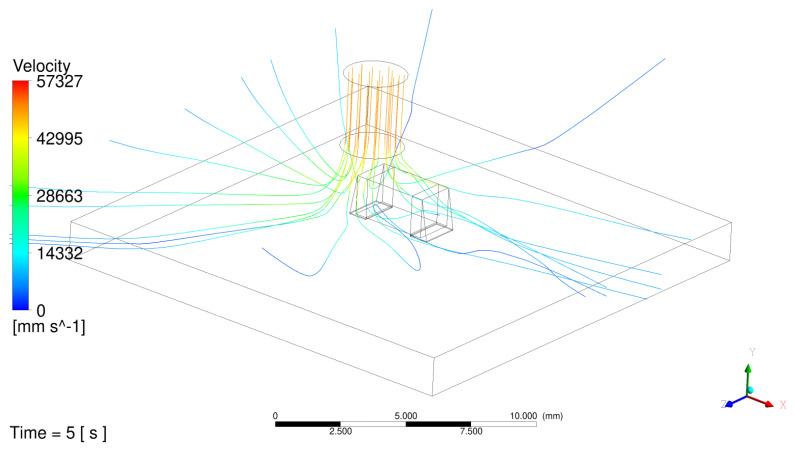
CFD model showing streamlines released from inlet colored by velocity magnitude in mm/s on 1206 MLCC.

Figure 10 displays streamlines released from the CFD velocity inlet boundary after 5 s, illustrating the flow path from inlet to exit boundaries, including impingement on the MLCC and localized recirculation. It can be observed that the flow follows the contours of the solder shape on the left terminal, but separates over the right terminal, leading to a region of recirculating flow on that side.

**Figure 11:**
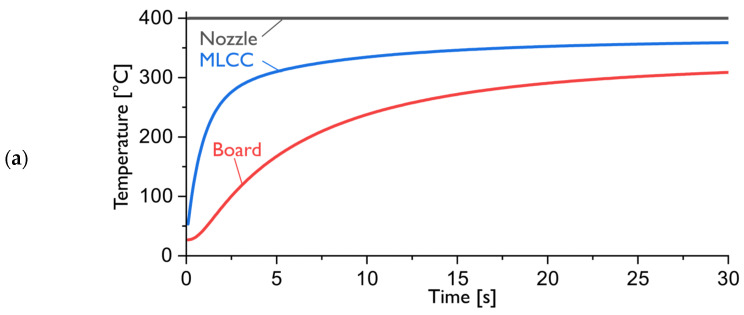

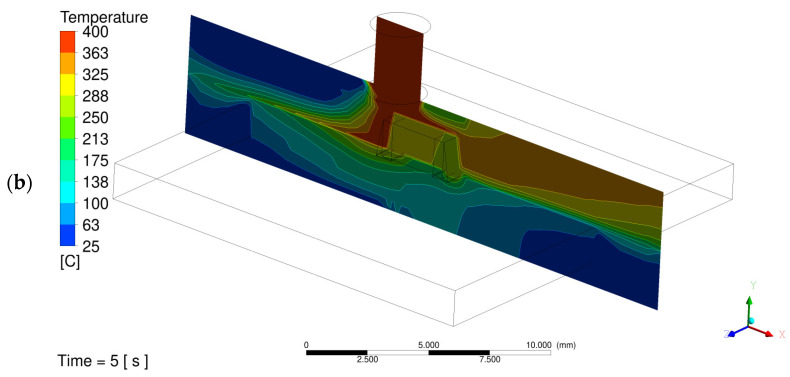
Evolution of temperature during the desoldering process: (**a**) Average board surface temperature and average MLCC body temperature; (**b**) CFD temperature contours on central xy-plane in CFD model after 5 s of heating at 400 °C.

Figure 11 depicts the temperature evolution during the desoldering process. Figure 11a shows the temporal temperature evolution. The average temperature of the MLCC ceramic body (blue line) and the average surface temperature of the board (red line) are plotted over time. Figure 11b shows the spatial temperature evolution. The temperature contours on the component and board surfaces are depicted for a time of 5 s after the heating with the reworking tool has started. The graphic highlights the temperature differentials across the assembly leading to induced stresses.

**Figure 12:**
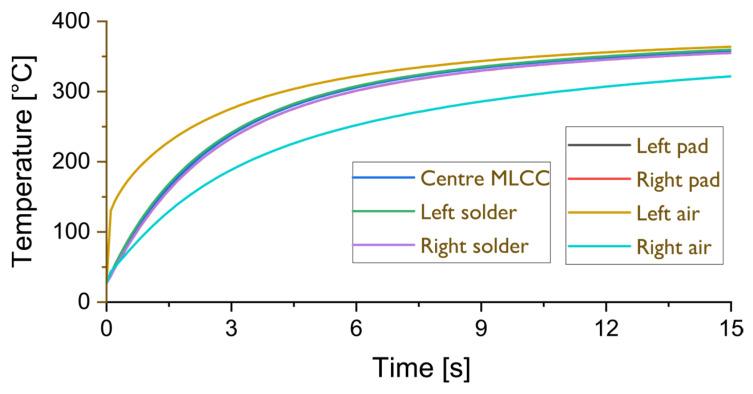
Temperature evolution of the component during desoldering: CFD-simulated time-temperature development at 7 points within and around the 1206 MLCC component.

The diagram in Figure 12 gives more detailed information for the average MLCC body temperature plotted in Figure 11a. The temperatures for the center of the MLCC (blue line), the left (green line) and right solder joint (purple line), and the temperature for the left (black line) and right pad (red line) on the PCB are plotted over time together with the air temperatures on the left (yellow line) and right side (turquoise line) of the MLCC.

**Figure 13:**
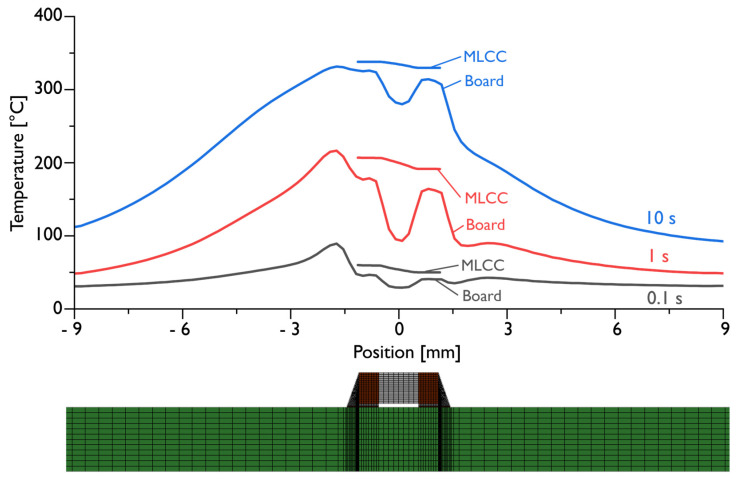
Spatial temperature distributions across the MLCC component and the board at the times of 0.1 s (black lines), 1 s (red lines), and 10 s (blue lines).

Figure 13 shows the spatial temperature evolution as a diagram. Depicted are the temperature distributions across the MLCC component and the board at the times of 0.1 s (black lines), 1 s (red lines), and 10 s (blue lines) after the heating with the reworking tool has started.

**Figure 14:**
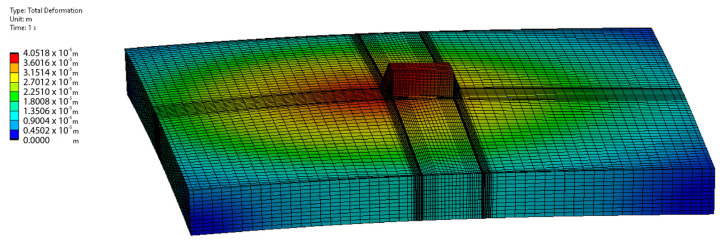
Evolution of deformation of the MLCC on PCB assembly: FEM-simulation-calculated displacement contours on MLCC component and PCB at a time of 1 s after the heating with the reworking tool has started. The maximum deformation occurs on the left side of the MLCC component, where the hot gas jet impinges on the PCB.

The resolved temperature fields from CFD simulations were used as input for a static mechanical assessment of thermomechanical performance. Figure 14 shows the displacement of each component, both suggesting a symmetrical distribution of stress and displacement across the component, potentially indicating that the thermal conductivity is sufficient to render the temperature differences throughout the component as a negligible factor in the accumulation of stresses.

**Figure 15:**
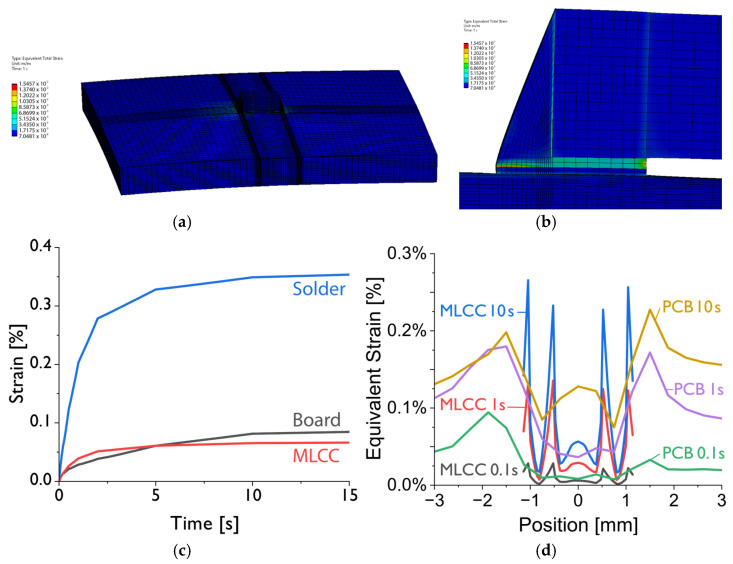
Simulated strain evolution during the desoldering process: (**a**) strain contours at a time of 1 s on the entire model; (**b**) strain contours of the solder joint zone; (**c**) comparison of average strain in the MLCC component, the solder joints, and the board; (**d**) spatial strain distribution in the MLCC and the board for the times of 0.1 s, 1 s, and 10 s.

Figure 15 shows the calculated strains. A contour plot of the strains across the entire model is depicted in Figure 15a, while Figure 15b shows a detailed magnification of this contour plot in the area around the solder joint. The diagram in Figure 15c shows the strain evolution over time in the solder joint (blue line), the board (black line), and the MLCC (red line). The spatial strain distribution in the MLCC and the board is displayed in the diagram in Figure 15d for the times of 0.1 s, 1 s, and 10 s.

**Figure 16:**
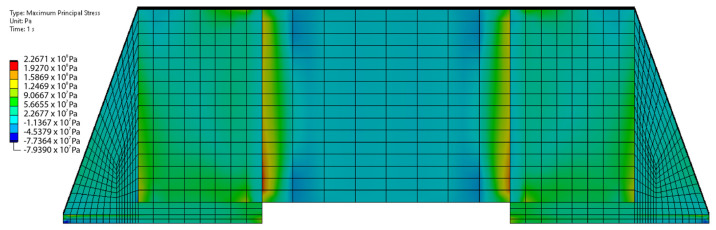
Evolution of mechanical stresses within the component: FEM-simulation-calculated maximum principal stresses on MLCC component at a time of 1 s after the heating with the reworking tool has started. The top value of these stresses is situated in the transition between the termination and the ceramic BiTiO_3_ body of the MLCC.

Figure 16 presents the contours of the maximum principal stress on the MLCC component, the solder, and the respective pads at a time of 1 s after the heating with the reworking tool has started. The peak value of the maximum principal stress occurs in the transition between the termination and the ceramic body of the MLCC.

## 4. Discussion

The comparison between the results of the temperature measurement (Figure 9) demonstrates the difficulties of the current experimental methodology in registering temperatures during a reflow soldering process. Although the temperature profiles were expected to be similar, it shows that the temperatures recorded with the FR-811 rework station rise (Figure 9a) much slower than those recorded with the 852-A rework station (Figure 9b,c). This difference is believed to be caused by the external temperature control loop of the FR-811 rework station. While the internal temperature control of 852-A rework station sets the heater in the pistol immediately to the target temperature of 400 °C, the FR-811 temperature control algorithm is designed to adjust the heating power to the object being soldered. Therefore, smaller components, like the 0805 and 1206 MLCCs used in this study, will be heated more moderately, to avoid severe thermal stresses. This feature makes the FR-811 rework station a less suitable device for experimentation because it is very difficult to model the heating behavior of the device. The strong differences between the simulated temperature profile (black dashed line) and the monitored profile (blue line) in Figure 9a is supposed to be caused by the temperature control algorithm of the FR-811 station. This was the reason to conduct another set of experiments using the 852-A rework station.

If the monitored temperatures from the experiments with the 852-A rework station are compared (Figure 9b vs. Figure 9c), it shows that while the temperature profiles for the MLCC body temperature (black lines) are very similar, there is a strong difference in the temperature profiles for the board (red lines). This might be explained by the different ways the temperature measurement is organized. While the thermocouple touches the board directly from the top, at the same moment it also has contact with the hot jet coming from the nozzle (see streamlines depicted in Figure 10), which provides additional heating of the thermocouple’s legs. In contrast, the integrated PT-100 sensor takes the temperature indirectly from the board via the ceramic body of the SMT-resistor. Since it is placed on the bottom side of the resistor, it has no direct contact with the hot jet. For that reason, the board temperature recorded by the PT 100 sensor might be lower than that recorded by the thermocouple.

If the experimental gained body temperature of the MLCC (blue lines) from the diagrams in Figure 9b,c, are compared to the dashed black line in these diagrams (which represents the MLCC body temperature calculated by simulation), a rough agreement between the experimental and simulation results can be observed. However, it needs to be noted that the simulation assumed a flow speed from the FR-811 rework station, which is somewhat higher compared with that of the 852-A.

One of the remarkable results that can be read from the diagrams depicted in Figure 12 and Figure 13 is the temperature homogeneity across the MLCC during the desoldering, although the hot gas jet was set intentionally asymmetrically only to one side of the component, in order to represent worst-case scenarios that may occur in manual operation or due to misalignment of automated rework equipment. While the temperature difference in the surrounding gas is as high as 150 K, the temperature difference from the right termination of the MLCC to its left termination never exceeds 20 K. The tightness of the temperature contours across the component are well illustrated by the temperature contour in Figure 11b and the diagram in Figure 12, where the temperature inside the left and right copper pads, at the center of the MLCC, inside the left and right solder joints and the first mesh points inside the air on the left and right side of the component, are labeled “left air” and “right air”, respectively. Here, one can observe that, in summary, the temperature increases steadily in unison throughout the component with variations smaller than 20 K but that, owing to the large recirculation region on the right side of the MLCC, there exists a large temperature difference between the two sides of the component.

Therefore, the thermomechanical stress that is built up within the capacitor is supposed to be very moderate, because from the simulation results, it can be concluded that the lower coefficient of thermal expansion (CTE) combined with the higher coefficient of thermal conductivity (CTC) compensates the mismatch to the reverse properties of the PCB (high CTE, low CTC). But, still, the major contribution comes from thermomechanical stresses due to the mismatch in thermal expansion between the board and the MLCC, as the results in the diagrams of Figure 15 show.

Nonetheless, very high stresses (close to 200 MPa) can build up within the transition region from the termination to the BaTiO3 ceramic body of the MLCC component (see Figure 16). However, the employed material model for the solder (see Table 3), which was taken as a compromise for the current model development at the concept stage (to save computational time), has many shortcomings and oversimplifies the complex mechanical behavior of the solder. Thus, the simulated values might eventually overestimate the maximum principal stresses within the MLCC component during the desoldering process. However, it is believed that the simulated mechanical stresses at times of less than 1 s are still somewhat realistic. Since the solder temperature at 1 s is still below its melting point, the absence of a melting/solidification approach will not affect the validity of the solder modeling in an unsupportable way.

## 5. Conclusions

Experiments and simulations were carried out in order to gain a better understanding of the thermomechanical stresses that build up during desoldering processes in the rework of printed circuit boards. The results of the study show two things: (a) With regard to the current experimental techniques, there are some methodological difficulties in monitoring temperatures accurately during reflow soldering with hot air pistols; (b) In order to simulate the effects of the thermal loading, a framework of computational fluid dynamics and thermomechanical models can calculate more detailed information about mechanical stresses that build up during the rapid reflow desoldering process.

For the given example of the desoldering of an MLCC component, it can be concluded from the simulations results that the major contribution to mechanical stresses within the ceramic capacitor comes from the mismatch in thermal expansion between the PCB and the MLCC. The results also show that, even with unfavorable soldering conditions, the temperature gradients along the MLCC component shall be rather small. However, the calculated internal stresses within the MLCC are still critical. The validation of the accuracy of simulation results needs the development of better experimental methods to capture more process data.

## Figures and Tables

**Table 1:** MLCC components used for experiments.

MLCC	Size	Dimension (L × W × T) (mm)	Dielectric
KEMET C0805C684K5RACTU	0805	2 × 1.25 × 1.25	X7R
KEMET C1206C474K5RACTU	1206	3.2 × 1.6 × 0.9	X7R
KYOCERA AVX 08055G104ZAT2A	0805	2.01 × 1.25 × 1.02	Y5V
KYOCERA AVX 12065G105ZAT2A	1206	3.20 × 1.60 × 1.27	Y5V

**Table 2:** Material data for CFD simulations.

Material	Thermal Properties		
	Density (kg/m^3^)	K (W/m·K)	Cp (J/kg·K)
MLCC	3474.8	13.739	772.09
Terminals	8942.5	396.58	383.48
PCB	1944.5	0.4899	962.56
Solder	7429.7	58.2	223.64
Pads	8942.5	396.58	383.48

**Table 3:** Material data for FEM simulation.

	CTE (ppm/K)	Young’s Modulus (GPa)	Poisson’s Ratio	Tensile Ultimate Strength (GPa)	Tensile Yield Strength (MPa)	Bilinear Tangent Modulus (MPa)
MLCC	9.04	246	0.2392	20.0	-	-
Terminals	16.7	126	0.345	34.3	-	-
PCB	14.7	24.4	0.1649	29.8	-	-
Solder	24.0	29.9	0.3899	-	38	353
Pads	16.7	126	0.345	25.1	-	-

## Data Availability

Data are contained within the article.
